# Cell-Free DNA Based Next-Generation Sequencing Does Not Differentiate Between Oligoprogression and Systemic Progression in Non-Small Cell Lung Cancer Patients Treated with Immune Checkpoint Inhibitors—An Explorative Study

**DOI:** 10.3390/ijms26168087

**Published:** 2025-08-21

**Authors:** Pim Rozendal, Hanneke Kievit, Paul van der Leest, Idris Bahce, Michiel Pegtel, Harry J. M. Groen, Léon C. van Kempen, T. Jeroen N. Hiltermann, Ed Schuuring

**Affiliations:** 1 Department of Pathology, University Medical Center Groningen, University of Groningen, 9713 GZ Groningen, The Netherlands; p.rozendal@umcg.nl (P.R.); p.vd.leest@nki.nl (P.v.d.L.); leon.van.kempen@mumc.nl (L.C.v.K.); 2 Department of Pulmonary Medicine, University Medical Center Groningen, University of Groningen, 9713 GZ Groningen, The Netherlands; h.kievit@umcg.nl (H.K.); h.j.m.groen@umcg.nl (H.J.M.G.); 3 Department of Pathology, Cancer Center Amsterdam, Amsterdam UMC, VU University, 1081 HV Amsterdam, The Netherlands; i.bahce@amsterdamumc.nl (I.B.); d.pegtel@amsterdamumc.nl (M.P.)

**Keywords:** non-small cell lung cancer (NSCLC), oligoprogression, immune checkpoint inhibitors (ICIs), circulating tumor DNA (ctDNA), next-generation sequencing (NGS)

## Abstract

Immune checkpoint inhibitors (ICIs) are a key treatment for advanced non-small cell lung cancer (NSCLC), but most patients will ultimately experience disease progression due to acquired resistance to ICI. Clinically, it is relevant to differentiate between systemic progression (SP) and oligoprogression (OP). Following SP, ICI treatment is usually discontinued, while in OP, patients are preferably treated with local ablative treatment with continuation of the ICI treatment. However, with progressive disease, it remains difficult to differentiate between true OP or SP. Circulating tumor DNA (ctDNA) analysis provides an accurate real-time reflection of the tumor burden. It remains elusive if ctDNA abundance and/or dynamics can discriminate between OP and SP. Therefore, the aim of this exploratory cohort study is to evaluate whether the sequential molecular tumor profiling of ctDNA is suitable for discriminating between true OP and SP in advanced NSCLC. Patients with stage III/IV NSCLC showing progression after ≥3 months of ICI were included. OP was defined retrospectively by RECIST response ≥ 6 months after local treatment and continued ICIs. Serial plasma samples were analyzed using the AVENIO ctDNA Expanded NGS assay targeting 77 cancer-related genes. Twenty patients (6 OP, 14 SP) were included. Somatic alterations were detected in 16 patients (median 4 mutations). No significant differences in baseline ctDNA levels, changes at progression, or mutation patterns were observed between OP and SP. Although ctDNA levels generally decreased early after the start of ICI treatment, and were increased at disease progression, mutational profiles of the 77 genes using the AVENIO Expanded ctDNA panel did not distinguish OP from SP.

## 1. Introduction

Treatment with immune checkpoint inhibitors (ICIs) in advanced non-small cell lung cancer (NSCLC) patients without targetable genetic alterations demonstrate long-lasting therapy response and overall survival (OS) in a subset of patients [1]. However, programmed death ligand 1 (PD-L1) expression—the only biomarker approved for clinical practice—and other candidate predictive biomarkers such as tumor mutational burden (TMB), and HLA expression insufficiently discriminate tumor responders from non-responders [2,3,4,5,6]. Moreover, after initial response to treatment, the disease will evolve in most patients to progressive disease, that can either be systemic progression (SP) or oligoprogression (OP). OP is defined as the growth or new appearance of a limited number (up to 5) of metastases under systemic treatment [7,8]. These limited number of metastases must be suitable for local ablative treatment such as surgery or ablative radiotherapy. OP differs clinically from SP by the limited number of progressing lesions and the continuation of systemic treatment combined with local radical treatment of the metastatic side (s), which is associated with prolonged survival compared to SP [9,10,11]. In clinical practice, the assessment of OP often remains challenging, as it may be reclassified as SP shortly after local radical treatment, retrospectively indicating that it should have been classified initially as SP. Misclassification of OP leads to unnecessarily prolonged ICI treatment of patients with expensive, ineffective drugs [12,13]. Therefore, biomarkers are needed to distinguish true OP from SP in patients treated with ICI.

In the context of immunotherapy, it is known that longitudinal circulating tumor DNA (ctDNA) analysis has potential clinical value to predict and monitor response to therapy in advanced NSCLC patients [12,13,14,15]. Multiple studies reported that an early decrease in ctDNA levels is associated with prolonged ICI therapy response, whereas an absence of a decrease in ctDNA levels correlates with both a lower progression-free survival (PFS) and OS [13,16,17,18]. Although it was observed that ctDNA levels decreased in metastatic NSCLC patients diagnosed with OP after stereotactic body radiation therapy (SBRT) with continuation of systemic therapy, it remains unclear whether ctDNA dynamics can reliably distinguish OP from SP [10]. NGS-based ctDNA monitoring can provide a real-time overview of tumor burden and mutational clonal evolution and might be suitable as a tool to stratify between the two progression phenotypes.

We hypothesize that baseline ctDNA levels and ctDNA dynamics during treatment may reflect a better and more accurate method to differentiate between true OP and SP as compared to response evaluation criteria in solid tumors (RECIST 1.1) by computed tomography (CT) scans [13,15,19]. The AVENIO ctDNA Expanded NGS assay covering 77 clinically relevant genes was used on plasma samples to assess the changes in ctDNA levels and/or mutational profiles during treatment.

## 2. Results

### 2.1. Patient and Sample Characteristics

**Figure 1:**
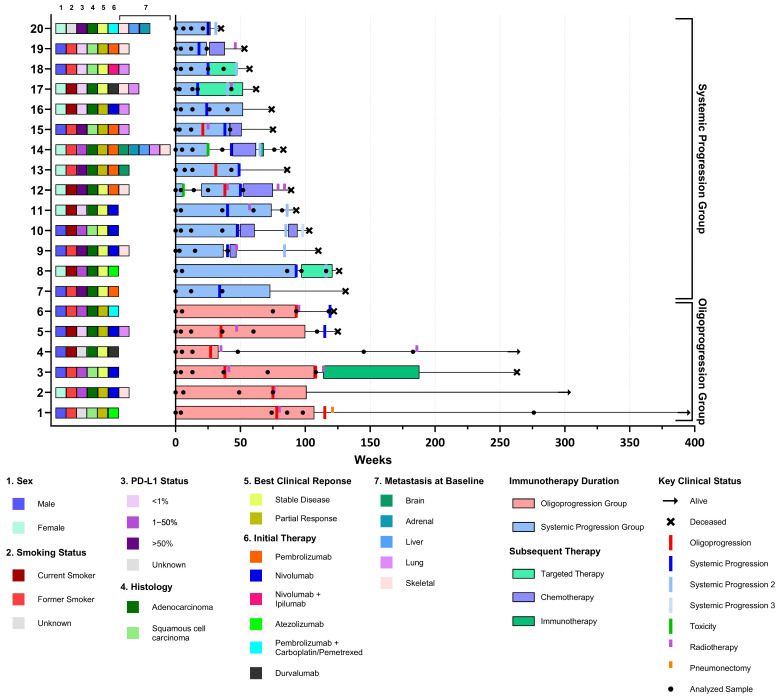
Swimmer plot of response. Swimmer plot showing response and duration to initial treatment with immunotherapy and subsequent therapy. All individual patients are displayed on the *y*-axis, ordered by patient group and overall survival. Indicated in each plot are the analyzed samples of each patient, moments of OP and SP. Key clinical features are indicated for each patient and survival status is presented at the end of each plot (Table 1).

Twenty patients (eighteen with stage IV and two patients with stage III after chemoradiotherapy in an adjuvant setting, receiving ICI) were selected. Six patients were retrospectively classified as OP and 14 patients as SP. Patient characteristics are summarized in Table 1 and Figure 1. All patients were treated with PD-1 or PD-L1 inhibitors, one patient received a combination with anti-CTLA4 therapy and two patients a combination with chemotherapy. Half of the patients received the ICI as first-line therapy. Sixteen patients (80%) discontinued ICI treatment due to progression, three patients after treatment of two years without SP, and one patient stopped after five months due to toxicity (Figure 1).

All six patients with OP received local ablative treatment, mostly patients had one location of OP. Of the patients with SP, three patients were treated with local ablative treatment during ICI treatment as well, but had systemic progression within 6 months (Table 1 and Supplementary Table S1). These patients were treated with ICI for an additional 12–18 weeks after local ablative treatment (Supplementary Table S1).

**Figure 2:**
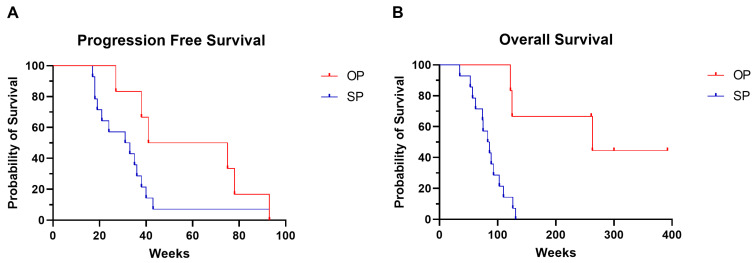
Kaplan–Meier curve showing (**A**) progression-free survival and (**B**) overall survival of OP and SP patient group.

Median PFS of the whole cohort was 37 weeks (range 17–93 weeks), median OS 98 weeks (range 35 weeks—not reached) and median duration of treatment was 46 weeks (range 15–105 weeks). Both OS (262 weeks vs. 85 weeks, *p* = 0.002) and PFS (58 weeks vs. 34 weeks, *p* = 0.04) were significantly longer in the OP group (Table 1, Figure 2). Patients with SP were more likely to have a higher metastatic load when looking at both the total number of metastases and the metastatic organs involved at baseline, although these differences were not significant. Based on the clinical–pathological and treatment response data, OP only differed significantly from SP by survival (Table 1).

### 2.2. ctDNA Profiling to Discriminate Between True OP and SP

Selection of patients in this study required the availability of serially collected plasma samples during treatment until (oligo)progression (Figure 1). In total, 95 samples were collected and analyzed, averaging 5 samples per patient (range 3–6).

**Figure 3:**
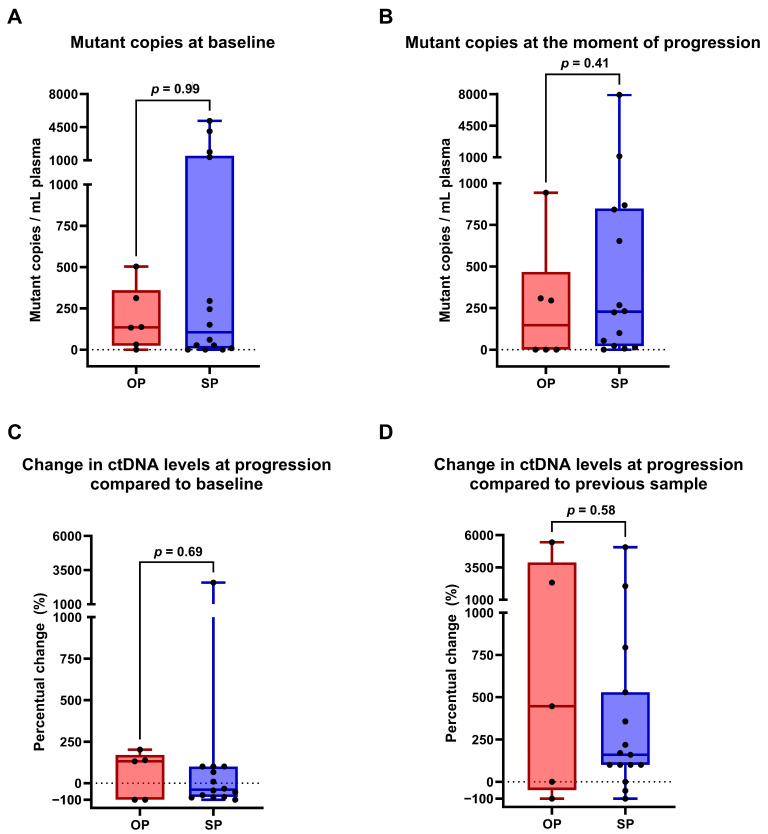
Comparison of total mutant ctDNA levels between patients with OP (red) and without OP (blue). Barplots depicting differences in total levels of mutant ctDNA. (**A**) At baseline, ranging from 0 to 503 (median 135) copies/mL plasma in OP and from 0 to 5169 (median 106) copies/mL plasma in SP (*p* = 0.99) and (**B**) at progression ranging from 0 to 943 (median 148) copies/mL plasma in OP and ranging from 0 to 7914 (median 228) copies/mL plasma in SP (*p* = 0.41). (**C**,**D**) Barplots depicting changes in ctDNA levels at OP or SP compared to either baseline (*p* = 0.69) (**C**) or the sample measured before the moment of (oligo)progression (*p* = 0.58) (**D**). Mann–Whitney test *p*-values < 0.05 are considered significant.

In order to evaluate whether plasma-based molecular profiling is able to discriminate between OP and SP in advanced NSCLC treated with ICI, we first compared ctDNA levels at baseline and at the moment of OP or SP. For patients with OP, ctDNA at baseline was detected in 5/6 patients (83%), at progression ctDNA was detected in 3/6 patients (50%). For patients with SP, ctDNA was detected at baseline in 11/14 patients (79%), and at progression in 13/14 patients (93%) (Supplementary Table S2A,B). ctDNA levels at baseline or at progression did not differ between patients with either OP or SP (Figure 3A,B).

Initially, 3/5 patients (60%) in the OP group and 9/11 (82%) in the SP group with detectable baseline ctDNA showed a decrease in ctDNA levels at the first moment of treatment evaluation (Supplementary Figure S1). Next, we assessed changes in ctDNA levels between baseline and progression in plasma samples, comparing OP or SP. Changes in ctDNA levels ranged from −100 to +202% (median 115%) in OP and from −100% to 2583% (median −39%) in SP (Figure 3C). Changes between the sample collected shortly prior to progressive disease and progression showed variation from −100% to 5447% (median 446%) in OP and from −100% to 2063% (median 160%) in SP (Figure 3D). The changes in ctDNA levels during treatment were not different in OP compared to SP.

### 2.3. Comparative Analysis of Somatic Variants in OP Versus SP

Molecular mutational profiling of all 95 plasma samples from 20 patients across multiple timepoints revealed that at least one somatic variant was detected in 19 out of 20 patients (95%) at one or more timepoints. Across all analyzed samples, between 0 and 8 variants were detected per sample, with a median of 4. Both at baseline and progression, mutations were found in 16/20 patients (80%). An overview of the various somatic variants found in each patient during treatment is shown in Supplementary Figure S1. Additionally, an overview of all observed somatic variants is shown in Supplementary Table S3.

**Figure 4:**
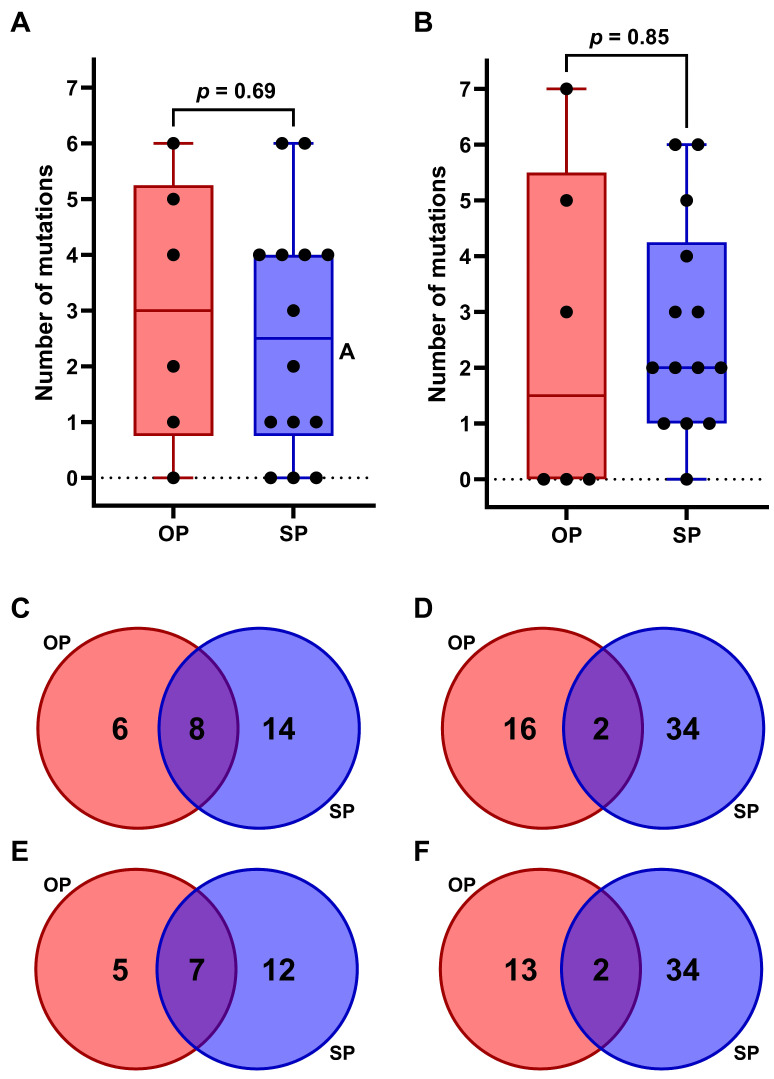
Comparison of number of variants found in patients with OP (red) or SP (blue)**.** (**A**) Barplot depicting number of mutations found at baseline (*p* = 0.69) (**B**) Barplot depicting number of mutations found at OP or systemic progression (*p* = 0.85). *p*-values < 0.05 are considered significant. (**C**,**D**) Venn diagram showing the overlap and difference in unique variants between OP and SP at baseline on gene level (**C**) and variant level (**D**–**F**). Venn diagram showing the overlap and difference in unique variants between OP and SP at progression on gene level (**E**) and variant level (**F**).

We evaluated whether the number of variants found in OP and SP differed. At baseline, different somatic variants ranged from 0 to 6 (median = 3) in OP and from 0 to 6 (median = 2.5) in SP patients. At progression, the number of somatic variants ranged from 0 to 7 (median = 1.5) in OP and from 0 to 6 (median = 2) in SP patients. Overall, the number of mutations did not differ between OP and SP (Figure 4A,B).

Next, we evaluated whether OP and SP were associated with the presence of specific mutations. At baseline, in the OP group, the six cases had 18 unique variants in 14 genes, while in the SP group, the fourteen cases had 36 unique variants in 22 genes (Figure 4C,D) (Supplementary Table S3). At progression in OP patients, 15 unique variants were found across 12 genes, while in SP, 38 unique variants were detected in 19 genes (Figure 4E,F). Three genetic variants were found only in the OP group at progression, specifically in the *TP53* and *PTCH1* genes. These variants were unique to patients 2, 3, and 5 (see Supplementary Figure S1 and Table S3). Additionally, five unique variants were exclusively identified at the time of SP in the SP group in the *KRAS*, *EGFR*, *TP53*, *KEAP1*, and *MTOR* genes, found in patients 7, 10, 12, and 18.

Somatic variants found at progression could be associated with treatment resistance. In the cohort of 20 patients, 53 variants were found across 24 genes at progression (Supplementary Table S3). At OP, 9 pathogenic variants were found, and 6 variants of unknown significance (VUS) were identified. At SP, 20 pathogenic variants were found and 21 VUS. In our cohort, a *KEAP1* or *STK11* variant was detected in six patients (OP-group *n* = 2, SP-group *n* = 4), with the respective variant being observed at the time of progression in five of these patients (Supplementary Table S3).

## 3. Discussion

ICI treatment and targeted therapy in NSCLC have resulted in prolonged tumor control and survival. Upon progression, it is important to recognize OP and SP to determine the appropriate treatment: continuing ICI with selected ablative therapy for local control in case of OP or discontinue ICI in case of SP. To our knowledge, this is the first exploratory study assessing the applicability of targeted ctDNA sequencing to discriminate between OP and SP in patients receiving ICI treatment. In this study, we found that the majority of patients showed typical ctDNA responses to ICI therapy with an initial ctDNA decrease followed by an increase at the moment of progression. However, we could not discriminate between OP and SP based on ctDNA levels and/or changes, although ctDNA was more prevalent in SP cases compared to OP at time of disease progression (93% vs. 50%). No significant difference in ctDNA patterns between OP and SP were found. We were not able to distinguish between OP and SP based on ctDNA assessment with a targeted NGS panel.

As expected, patients with OP predominantly demonstrated better OS than patients with SP [11]. The patient groups differ slightly, with mainly the number of metastases and organs involved at baseline being higher in the SP group, suggesting a higher tumor load. Also, SP patients had more metastases in organs associated with poorer prognosis (i.e., liver, brain, and bone) [5]. Those differences were not significant, probably due to the small sample size, but may contribute to poorer overall survival. Four of six OP cases (67%) received ICI as ≥2nd-line treatment, with none having a PD-L1 score of >50%, which is the only approved biomarker to select patient for ICI treatment [5]. In half of the cases the primary tumor, not a metastasis, was irradiated at the moment of OP. This is in line with Brown et al. with ICI as first-line treatment—who showed irradiation of the primary tumor in 21.4% of the cases, being the site of OP in 37.1% of their cases [9].

Semenkovich et al. showed in a cohort of oligometastatic NSCLC patients that no detectable ctDNA prior to radiotherapy (RT) was associated with a better PFS and OS after RT [20]. A similar trend was shown in preliminary data from Rui Fu et al. [21]. They found that NSCLC patients treated with targeted therapy or ICI (24.3% of the cohort) had a better prognosis after local consolidative treatment when ctDNA was absent at the time of induced OP. This subgroup, identified as molecular oligometastatic disease (MOD), showed improved PFS following local consolidative treatment compared to patients with non-MOD patients, which were ctDNA positive at the moment of OP. In our OP population, MOD was seen in patient six only, while three patients showed an increase in total mutant copies/mL plasma of ≥1 mutation. Two of these three showed a decrease again after local ablative therapy, suggesting an effective ablative treatment for this specific mutation. Additionally, patient 9 in the SP group had MOD. Within our cohort, MOD was not a determining factor for improved OS. Arrieta et al. found that 56% of NSCLC patients treated with EGFR-TKIs tested positive for ctDNA at the time of oligoprogression [22]. Although these patients were treated with TKIs and not immunotherapy, this is comparable to our cohort, where only 50% of the patients had detectable ctDNA at the time of OP. So, absence of ctDNA at time of progression itself does not distinguish between OP and (the start of) SP.

Low ctDNA copy numbers may limit its effectiveness as a marker for clinical decision making. In three patients with ≤30 mutant copies mL/plasma, we did observe aberrant patterns with decreases to zero and subsequent increases without therapy change. However, in our cohort, the opposite was observed as well, in which a sudden increase in ctDNA was followed by a decrease to zero copies, indicating that mutations were possibly present below the limit of detection of the AVENIO ctDNA Expanded assay. This may complicate the interpretation of ctDNA measurements for therapy-response monitoring. As described previously, we only included variants with a variant read depth of ≥10 unless detected at multiple timepoints [23]. Our results indicate that in patients with low variant read numbers, ctDNA may be less reliable to use as a marker for clinical decision making. Moreover, Ding et al. demonstrated that the lowest concentration of ctDNA, determined by the median allele fraction, serves as a predictive indicator for PFS [24]. Furthermore, approximately 15–32% of NSCLC patients may not shed tumor DNA [25]. In our cohort, this was the case for only 5%, possibly indicating that part of non-shedding may be a sensitivity issue.

CtDNA measurements are a benchmark for predicting response to immunotherapy. Baseline and early-response ctDNA measurements show that the absence of ctDNA at baseline and early ctDNA decreases are indicative of superior PFS and OS [13,26]. However, they do not differentiate between OP and SP. Nonetheless, we do see in our cohort that patients with OP show a superior OS compared to SP patients. In line with previous studies, most patients with SP initially showed a decrease in ctDNA after the start of ICI followed by a ctDNA increase in (at least a part of the) mutations at time of progression [17,27]. In six patients, a *KEAP1* (*n* = 5) and/or *STK11* (*n* = 3) mutation was detected (Supplementary Table S3), of which five were considered pathogenic, two for OP and three for SP cases. It has been reported that *KEAP1* and *STK11* variants are associated with poorer responses to ICI, especially with lower PD-L1 scores of the tumor [28,29,30]. These variants do not seem to be specific for either group in our cohort.

This study has several limitations. First of all, it is a retrospective study, where patients were selected for ICI treatment with a tumor and clinical response for at least 3 months. We applied the RECIST criteria retrospectively on the CT scans, at which point patients turned out to have RECIST progression earlier than clinical progression. Secondly, this exploratory study has a small number of cases. In order to detect more subtle differences in ctDNA kinetics, a larger cohort is required. Furthermore, for the NGS analysis, we used the AVENIO ctDNA Expanded assay by Roche, which consists of a 77-gene panel containing actionable genes that are present in U.S. National Comprehensive Cancer Network (NCCN) guidelines and emerging cancer biomarkers [31]. Nonetheless, a more expansive NGS gene panel or whole-exome sequencing might have provided different insights and differentiate better between OP and SP or possible resistance mechanisms to ICI therapy, where we could not confirm our hypothesis.

## 4. Materials and Methods

### 4.1. Patient Inclusion and Sample Collection

NSCLC patients treated with ICI for stage III/IV disease, with serial plasma samples available, who initially responded to treatment with immune checkpoint inhibitors (ICIs) and subsequently experienced disease progression, were retrospectively selected for this study. Response was defined as partial response (PR) or stable disease (SD) for at least three months (i.e., at least two response evaluation scans) as assessed by diagnostic CT imaging according to RECIST v1.1. RECIST was scored retrospectively (by IB and HK and in cases with discrepancies also by TH). Tumor measurements were scored for the duration of ICI treatment. These patients were treated at the University Medical Center Groningen (UMCG) (*n* = 17) and the Amsterdam University Medical Center, VUmc (*n* = 3). For this study, we defined OP as limited progression (with a maximum of 5 different locations) that was treated with local ablative treatment and continuation of ICI for at least 6 months following primary progressive disease. Patients initially treated as OP but with subsequent SP < 6 months after local ablative treatment were classified as SP. This resulted in two groups: a SP group and an OP group.

Blood samples were prospectively drawn at baseline and serially during treatment. We selected samples for ctDNA analysis at several timepoints: before treatment initiation, after 1–2 months following the start of treatment, after at least three months of treatment or three months prior to confirmed progression, and at the time of confirmed disease progression, being either OP (first time, in case of more than one OP event) or SP.

Plasma samples collected in the UMCG and VUmc were processed as reported previously [23]. In short, plasma samples were collected in either EDTA blood-collection tubes (BCTs; Becton Dickinson, Franklin Lakes, NJ, USA) or Cell-Free DNA BCTs^®^ (Streck, Omaha, NE, USA). Blood-collection tubes were centrifuged at 820× *g* (EDTA) or 1600× *g* (Streck) for 10 min at room temperature. EDTA samples were processed within 4 h after venipuncture and Streck samples within 24 h. The supernatant was subsequently centrifuged at 16,000× *g* for 10 min at 4 °C. Cell-free plasma was stored in 1 mL fractions at −80 °C until circulating cell-free DNA (ccfDNA) extraction. For identification of clonal haematopoiesis (CH)-related variants, peripheral blood mononuclear cells (PBMCs) were isolated from EDTA BCTs and stored at −80 °C until DNA extraction. All patients provided written informed consent in the UMCG as part of the Oncolifes project, a hospital-based data-biobank for oncology [32]. Plasma samples from Amsterdam UMC were collected in accordance with the ethical standards of the institutional review board of Amsterdam UMC under protocol number 2017.333.

### 4.2. ccfDNA and gDNA Extraction

CcfDNA was extracted from 2 mL of cell-free plasma and eluted in 52 μL AVE elution buffer with QiaAMP Circulating Nucleic Acid Kit (Qiagen) according to the manufacturer’s recommendations [13,33,34,35]. Genomic DNA (gDNA) from the PBMCs was extracted using either a custom protocol (Supplementary Materials) or the Gentra Puregene Blood Kit (QIAGEN, Hilden, Germany) according to manufacturer’s recommendations with the following adaptation: Proteinase K was added to improve the digestion of the pellet and nuclease-free water was used to elute the isolated DNA [23]. DNA concentrations were determined using the Qubit dsDNA HS Assay (ccfDNA) or Qubit dsDNA BR Assay (PBMC DNA; both Thermo Fisher Scientific, Waltham, MA, USA).

### 4.3. ctDNA NGS Analysis and Interpretation

Molecular profiling was performed using the AVENIO ctDNA Expanded NGS assay (Roche, Basel, Switzerland) according to the manufacturer’s recommendations and as reported recently [23]. This pan-cancer panel consists of 77 clinically relevant genes described in the U.S. National Comprehensive Cancer Network (NCCN) guidelines as well as emerging cancer biomarkers specifically optimized for lung and colorectal cancer. A median input of 27.6 ng (range 5.7–51 ng) of ccfDNA and 50 ng (range 20–50 ng) of gDNA was used for the preparation of AVENIO libraries. Prior to adapter ligation, PBMCs were enzymatically fragmented using the KAPA Frag kit (Roche) followed by a bead cleanup using KAPA Pure beads (Roche) according to manufacturer’s recommendation with an incubation time of 30 min. Sequencing was performed on a NextSeq 550 (Illumina, San Diego, CA, USA). Resulting data were analyzed using AVENIO Oncology Analysis Software version 2.1.0 (Roche) with customized somatic variant filter settings [23]. Single nucleotide variants (SNVs) were excluded if the variant depth of the single nucleotide polymorphism (SNP) was <10 and only present at a single timepoint in the patient. Synonymous variants were excluded if the variant was not located in a splice region. CH-related variants were excluded if the variant was detected in both the PBMC sample and in the plasma sample of the matching patient. Our analyses were restricted to SNVs and InDels, due to low levels of ccfDNA in our cohort. CtDNA levels were assessed as the number of mutant molecules per mL plasma. The total number of mutant copies/mL plasma in each sample was calculated by adding up the mutant copies for every detected variant in that sample. The pathogenicity of the detected variants was determined by consulting the knowledge bases Franklin, cBioPortal and JAX-CKB.

### 4.4. Statistics

Descriptive statistics were used for patient characteristics. PFS was defined as the period between the start of ICI to the date of detection of OP or SP and OS as the time between the start of ICI and date of death or censoring at the date of 1 December 2024. Differences in ctDNA levels and patient characteristics on an ordinary scale were assessed with a Mann–Whitney test. Differences in categorical variables were analyzed with the Fischer’s exact test. Differences in number and type of mutations were determined with an unpaired *t*-test. SPSS 28 (IBM Corporation, Armonk, NY, USA) and GRAPHPAD PRISM 9.1.0 (GraphPad Software, San Diego, CA, USA) were used for all statistical analysis. A *p*-value of <0.05 was considered significant.

## 5. Conclusions

OP remains a clinical feature. In our exploratory cohort study, changes in ctDNA extracted from blood plasma during treatment tested by our targeted 77-gene NGS panel cannot differentiate between true OP and SP in NSCLC patients treated with ICI by panel-based NGS.

## Figures and Tables

**Table 1:** Patient characteristics.

	All Patients (*n* = 20)	Oligoprogression (*n* = 6)	Systemic Progression (*n* = 14)	*p*-Value
**Median age (range)**	67 (44–84)	70 (60–84)	64 (44–72)	0.09 ^1^
**Sex**				0.16 ^2^
Male	11 (55%)	5 (83%)	6 (43%)	
Female	9 (45%)	1 (17%)	8 (57%)	
**ECOG PS**				0.58 ^1^
0	10 (50%)	2 (33%)	8 (57%)	
1	8 (40%)	4 (67%)	4 (29%)	
2	1 (5%)		1 (7%)	
3	1 (5%)		1 (7%)	
**Histology**				1.00 ^2^
Adenocarcinoma	14 (70%)	4 (67%)	10 (71%)	
Squamous cell carcinoma	6 (30%)	2 (33%)	4 (29%)	
**Smoking status**				1.00 ^2^
Active smoker	8 (40%)	2 (33%)	6 (43%)	
Former smoker	11 (55%)	4 (67%)	7 (50%)	
Never smoker	0 (0%)	0 (0%)	0 (0%)	
Unknown	1 ((5%)	0 (07%)	1 (7%)	
**Current treatment**				Not applicable
Atezolizumab	2 (10%)	0 (0%)	2 (14%)	
Durvalumab	2 (10%)	1 (17%)	1 (7%)	
Nivolumab	7 (35%)	4 (67%)	3 (21%)	
Nivolumab/ipilimumab	1 (5%)	0 (0%)	1 (7%)	
Pembrolizumab	6 (30%)	0 (0%)	6 (43%)	
Pembrolizumab/carboplatin–pemetrexed	2 (10%)	1 (17%)	1 (7%)	
**Previous lines of therapies**				0.42 ^1^
0	10 (50%)	2 (33%)	8 (57%)	
1	5 (25%)	2 (33%)	3 (21%)	
2	4 (20%	2 (33%)	2 (14%)	
≥3	1 (5%)	0 (0%)	1 (7%)	
**PD-L1**				0.50 ^1^
<1%	6 (30%)	1 (17%)	5 (36%)	
1–50%	6 (30%)	3 (50%)	3 (21%)	
>50%	6 (30%)	0 (0%)	6 (43%)	
Missing	2 (10%)	2 (33%)	0 (0%)	
**Number of metastatic sites at start ICI treatment °**				0.19 ^1^
0	1 (5%)	0 (0%)	1 (7%)	
1	6 (30%)	3 (50%)	3 (21%)	
2	7 (35%)	2 (33%)	5 (36%)	
3	3 (15%)	1 (17%)	2 (14%)	
≥4	3 (15%)	0 (0%)	3 (21%)	
**Total number of metastases at start ICI treatment**				0.09 ^1^
<5	8 (40%)	4 (67%)	4 (29%)	
5–20	9 (45%)	2 (33%)	7 (50%)	
>20	3 (15%)	0 (0%)	3 (21%)	
**Metastases associated with poor survival before start ICI treatment**				
Brain	2 (10%)	0 (0%)	2 (14%)	0.34 ^1^
Liver	2 (10%)	0 (0%)	2 (14%)	0.34 ^1^
Bone	7 (35%)	1 (17%)	6 (43%)	0.27 ^1^
**Survival in weeks (range)**				
Median PFS	37 (17–93)	58 (27–93)	32 (17–93)	0.04 ^1^
Median OS	103 (35–NR)	262 (122–370)	85 (35–131)	0.002 ^1^
Median duration of ICI treatment	46 (15–105)	99 (31–105)	42 (15–90)	0.007 ^1^
Local ablative (radio)therapy during ICI treatment *	10 (50%)	6 (100%)	4 (29%)	Not applicable
Primary tumor	4 (20%)	3 (50%)	1 (7%)	
Metastasis	6 (30%)	3 (50%)	3 (21%)	

° per different organs (systems), multiple thoracic lymph nodes count as one organ and also multiple lymph nodes outside the thorax (for example, one patient with a cervical and an abdominal lymph node) were counted as one organ system. * For details, see Supplementary Table S1. ^1^ Mann–Whitney U test; ^2^ Fisher’s exact test.

## Data Availability

Data is available upon reasonable request.

## Supplementary Materials

The following supporting information can be downloaded at: https://www.mdpi.com/article/10.3390/ijms26168087/s1.

## Abbreviations

The following abbreviations are used in this manuscript:

Anti-CTLA4	Anti-Cytotoxic T-lymphocyte-associated protein 4
ccfDNA	Circulating cell-free DNA
CH	Clonal haematopoiesis
CT	Computed tomography
ctDNA	Circulating tumor DNA
ECOG	Eastern Cooperative Oncology Group
gDNA	Genomic DNA
ICI	Immune checkpoint inhibitors
InDels	Insertion/deletion mutation
MOD	Molecular oligometastatic disease
NCCN	National Comprehensive Cancer Network
NGS	Next-Generation Sequencing
NSCLC	Non-small cell lung cancer
OP	Oligoprogression
OS	Overall survival
RECIST	Response evaluation criteria in solid tumors
RT	Radiotherapy
PBMC	Peripheral blood mononuclear cells
PD-1	Programmed death 1
PD-L1	Programmed death ligand 1
PFS	Progression-free survival
PR	Partial response
SBRT	Stereotactic body radiation therapy
SD	Stable disease
SNP	Single-nucleotide polymorphism
SNV	Single-nucleotide variants
SP	Systemic progression
TMB	Tumor mutational burden
UMCG	University Medical Center Groningen
VUmc	location of Amsterdam University Medical Center
VUS	Variant of unknown significance
