# Supplementary material for: Cell-Free DNA Based Next-Generation Sequencing Does Not Differentiate Between Oligoprogression and Systemic Progression in Non-Small Cell Lung Cancer Patients Treated with Immune Checkpoint Inhibitors—An Explorative Study
Source: Int J Mol Sci. 2025 Aug 21;26(16):8087. doi: 10.3390/ijms26168087 (PMC12386807; doi:10.3390/ijms26168087)
Supplement: Supplementary file 1 [file ijms-26-08087-s001.zip › Supplementary File.pdf]

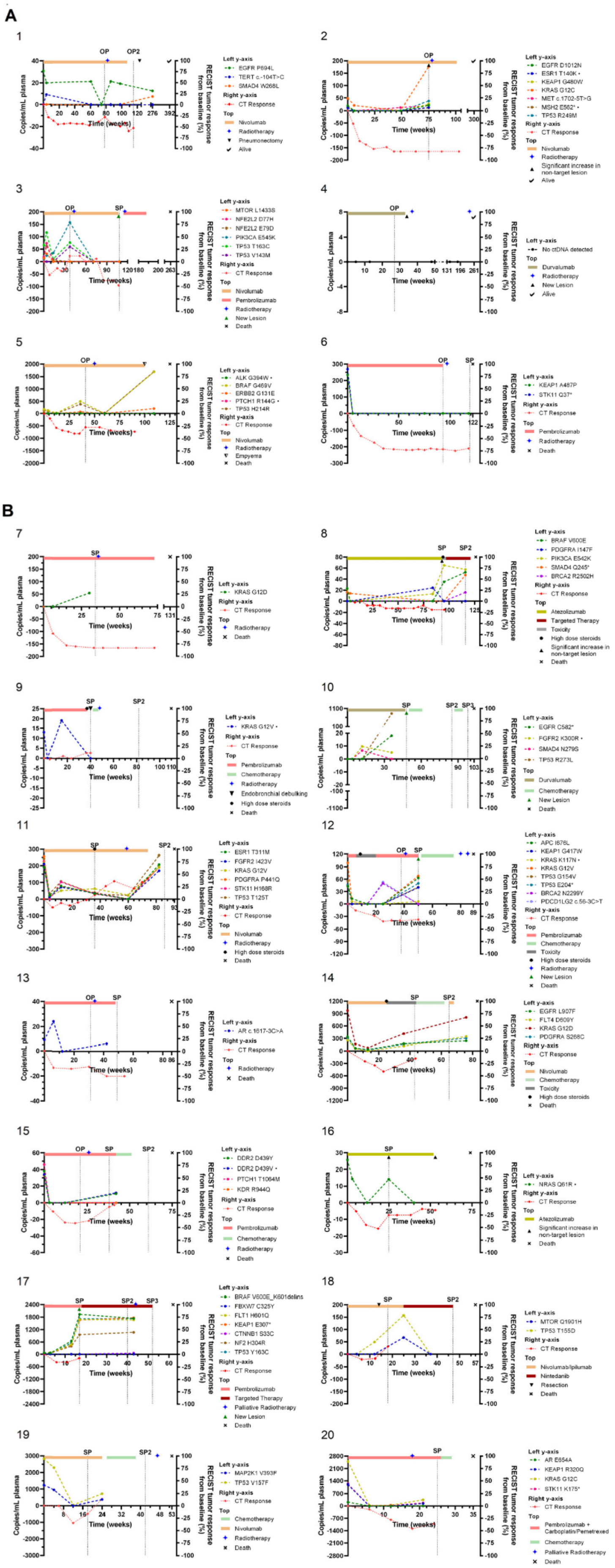

A
B
Copies/mL plasma
Time (weeks)
RECIST tumor response from baseline (%)
Left y-axis
Right y-axis
CT Response
Top
Nivolumab
Pembrolizumab
Durvalumab
Atezolizumab
Chemotherapy
Targeted Therapy
Toxicity
Radiotherapy
Palliative Radiotherapy
High dose steroids
New Lesion
Significant increase in non-target lesion
Pneumonectomy
Empyema
Endobronchial debulking
Resection
Nivolumab/Ipilumab
Nintedanib
Pembrolizumab + Carboplatin/Pemetrexed
No ctDNA detected
Alive
Death
OP
OP2
SP
SP2
SP3

Supplementary Figure S1: Detailed individual patient monitoring using ctDNA. Each profile combines mutant ctDNA levels with sum of target lesions change measured by CT scan (RECIST v1.1) of patients with OP (A) and SP (B). Patient numbers match with the numbers shown in Figure 1. Mutant ctDNA levels are plotted on the left y-axis. Variants marked with a dot had a variant depth < 10 but were detected at multiple timepoints within the same patient. Changes in tumor size (as sum of target lesions) are plotted on the right y-axis, in case of tumor progression due to new lesions or significant progression of non-target lesions this was indicated with a triangle at the moment of OP or SP. The bar on top shows which treatments the patients received over time, key clinical procedures, moment of OP, SP, SP2, SP3 (systemic progression for the respective second- or third time after subsequent systemic therapy) and final survival status. Each figure (1-20) depicts an individual patient, sorted by OS and patient grouping as described in table 2. Patients 12, 13, and 15 were initially treated as OP, but turned out to be SP. Patients 4 and 10 had no target lesions according to RECIST as they were treated with chemoradiotherapy preceding durvalumab treatment for stage 3 disease.

Supplementary Table S1: Local ablative treatment during ICI-treatment in study cohort

Patient	Site of progression	Local treatment	Duration to second progression* (weeks)
**OP**			
1	Primary tumor left lung	RT 10x3Gy	35 weeks
	2nd time OP: primary tumor	Pneumonectomy	Not reached
2	Rib metastasis (solitary)	RT 1x8Gy	Not reached
3	Primary tumor right lung	RT 17x3Gy	67
	2nd time OP: lymph nodes Naruke 8 +11/12L	RT 10x3Gy	Not reached/ died due to other cancer
4	Brain metastasis (solitary)	RT 1x 20Gy	149
	2nd time: 4 brain metastases	Gamma Knife	Not reached
5	Primary tumor left lung	RT 17x13Gy	68
6	Brain metastasis (solitary)	RT 24G in 3 fraction 3x8Gy	26
**SP cases initially treated as OP**			
12	lymph nodes paratracheal (1) and low cervical (1)	RT 5x4Gy	12
13	Primary tumor right lung	RT 8x7,5Gy	18
15	Left lung (nodule)	RT 1x 20Gy	17

RT = radiotherapy, Gy = gray

*Either systemic progression or oligoprogression occurring for the second time.

Supplementary Table S2A: Key ctDNA measurements in the OP group

	Baseline		OP			Sample before OP		
**Patient**	Mutant copies	Number of mutations	Mutant copies	Number of mutations	ctDNA change between baseline and OP	Mutant copies	Number of mutations	ctDNA change between this sample and OP
1	30,9	1	0	0	-100%	21	1	-100%
2	133,5	6	308,7	7	131%	12,7	1	2331%
3	137	5	295	3	115%	54	3	446%
4	0	0	0	0	N/A	0	0	N/A
5	312	4	943	5	202%	17	1	5447%
6	503	2	0	0	-100%	0	0	0%

Supplementary Table S2B: Key ctDNA measurements in the SP group

	Baseline		SP			Sample before SP		
**Patient number**	Mutant copies	Number of mutations	Mutant copies	Number of mutations	ctDNA change between baseline and SP	Mutant copies	Number of mutations	ctDNA change between this sample and SP
7	0	0	54	1	100%	0	0	100%
8	60	3	100	2	67%	37	2	170%
9	27	1	0	0	-100%	19	1	-100%
10	0	0	842	3	100%	16,3	2	5066%
11	1344	6	231	6	-83%	490	6	-53%
12	245	4	268	6	9%	103	2	160%
13	9	1	6	1	-33%	0	0	100%
14	2160	4	868	4	-60%	138	3	529%
15	151	4	23	2	-85%	0	0	100%
16	25,7	1	14,2	1	-45%	0	0	100%
17	295	6	7914	5	2583%	2485	5	218%
18	0	0	224	2	100%	0	0	357%
19	4714	2	1125	2	-76%	52	1	2063%
20	5169	4	653	3	-87%	73	2	795%

Supplementary Table S3: Overview of variants found in each patient

Patient	Variants detected in each patient							
1	*EGFR* p.P694L	*TERT* c.-104T>C	*SMAD4* p.W268L					
2	***TP53* p.R249M**	*EGFR* p.D1012N	*ESR1* p.T140K	***KEAP1* p.G480W**	***KRAS* p.G12C**	*MET* c.1702-5T>G	***MSH2* p.E562***	
3	***TP53* p.V143M**	***PIK3CA* p.E545K**	***TP53* p.T163C**	***MTOR* p.L1433S**	***NFE2L2* p.D77H**	***NFE2L2* p.E79D**		
4	No variants detected							
5	*PTCH1* p.R144G	*ALK* p.G394W	***BRAF* p.G469V**	*ERBB2* p.G131E	***TP53* p.H214R**			
6	*KEAP1* p.A487P	***STK11* p.Q37***						
7	***KRAS* p.G12D**							
8	***BRAF* p.V600E**	***PIK3CA* p.E542K**	*BRCA2* p.R2502H	*PDGFRA* p.I147F	***SMAD4* p.Q245***			
9	***KRAS* p.G12V**							
10	*EGFR* p.C582*	***TP53* p.R273L**	*FGFR2* p.K300R	*SMAD4* p.N279S				
11	*ESR1* p.T311M	*FGFR2* p.I423V	***KRAS* p.G12V**	*PDGFRA* p.P441Q	***STK11* p.H168R**	***TP53* p.T125T**		
12	*KEAP1* p.G417W	*APC* p.I676L	***KRAS* p.G12V**	***KRAS* p.K117N**	***TP53* p.G154V**	***TP53* p.E204***	*BRCA2* p.N2299Y	*PDCD1LG2* c.56-3C>T
13	*AR* c.1617-3C>A							
14	*EGFR* p.L907F	*FLT4* p.D609Y	***KRAS* p.G12D**	*PDGFRA* p.S268C				
15	*DDR2* p.D439Y	*DDR2* p.D439V	*KDR* p.R944Q	*PTCH1* p.T1064M				
16	***NRAS* p.Q61R**							
17	***BRAF* p.V600E_K601 delins**	*FLT1* p.H601Q	***KEAP1* p.E307***	*NF2* p.H304R	***TP53* p.Y163C**	*CTNNB1* p.S33C	*FBXW7* p.C325Y	
18	*MTOR* p.Q1901H	***TP53* p.T155D**						
19	*MAP2K1* p.V393F	***TP53* p.V157F**						
20	*KEAP1* p.R320Q	***KRAS* p.G12C**	***STK11* p.K175***	*AR* p.E654A				

Supplementary Table S3: Variants exclusively found at the moment of OP are marked red, variants found at OP and other timepoints are marked pink. Variants found exclusively at SP are marked dark blue, variants found at SP and other timepoints are marked light blue. Variants marked white were not detected at the moment of OP or SP. Variants in bold text are considered pathogenic or likely pathogenic.
