# Supplementary material for: Cell-Free DNA Based Next-Generation Sequencing Does Not Differentiate Between Oligoprogression and Systemic Progression in Non-Small Cell Lung Cancer Patients Treated with Immune Checkpoint Inhibitors—An Explorative Study
Source: Int J Mol Sci. 2025 Aug 21;26(16):8087. doi: 10.3390/ijms26168087 (PMC12386807; doi:10.3390/ijms26168087)
Supplement: Supplementary file 1 [file ijms-26-08087-s001.zip › Supplementary Material.pdf]

Supplementary Materials

Genomic DNA Isolation from PBMCs

After isolation of the PBMCs, 50 µl 10% SDS and 2.5 µl proteinase K (20 mg/ml) was added to the pellet and incubated overnight at 55°C. Next, 180 µl 6M NaCl and 700 µl of chloroform were added and subsequently mixed for one hour on a top-over rotor. Then the mixture was centrifuged for 30 min at 5500rpm at 4°C. From the upper phase, 500 µl was mixed with 500 µl isopropanol and centrifuged for 20 minutes at 20.000g at 4°C. The resulting pellet was washed with 500 µl 70% ethanol and centrifuged for 10 minutes at 20.000g twice. TE buffer was added to the pellet and incubated overnight on a roller. DNA was quantified using the Qubit dsDNA BR Assay Kit (Thermo Fisher Scientific, Waltham, MA, USA).
